# Supplementary material for: The conserved protein Seb1 drives transcription termination by binding RNA polymerase II and nascent RNA
Source: Nat Commun. 2017 Apr 3;8:14861. doi: 10.1038/ncomms14861 (PMC5382271; doi:10.1038/ncomms14861)
Supplement: Supplementary Information — Supplementary Figures, Supplementary Tables, Supplementary Methods and Supplementary References [file ncomms14861-s1.pdf]

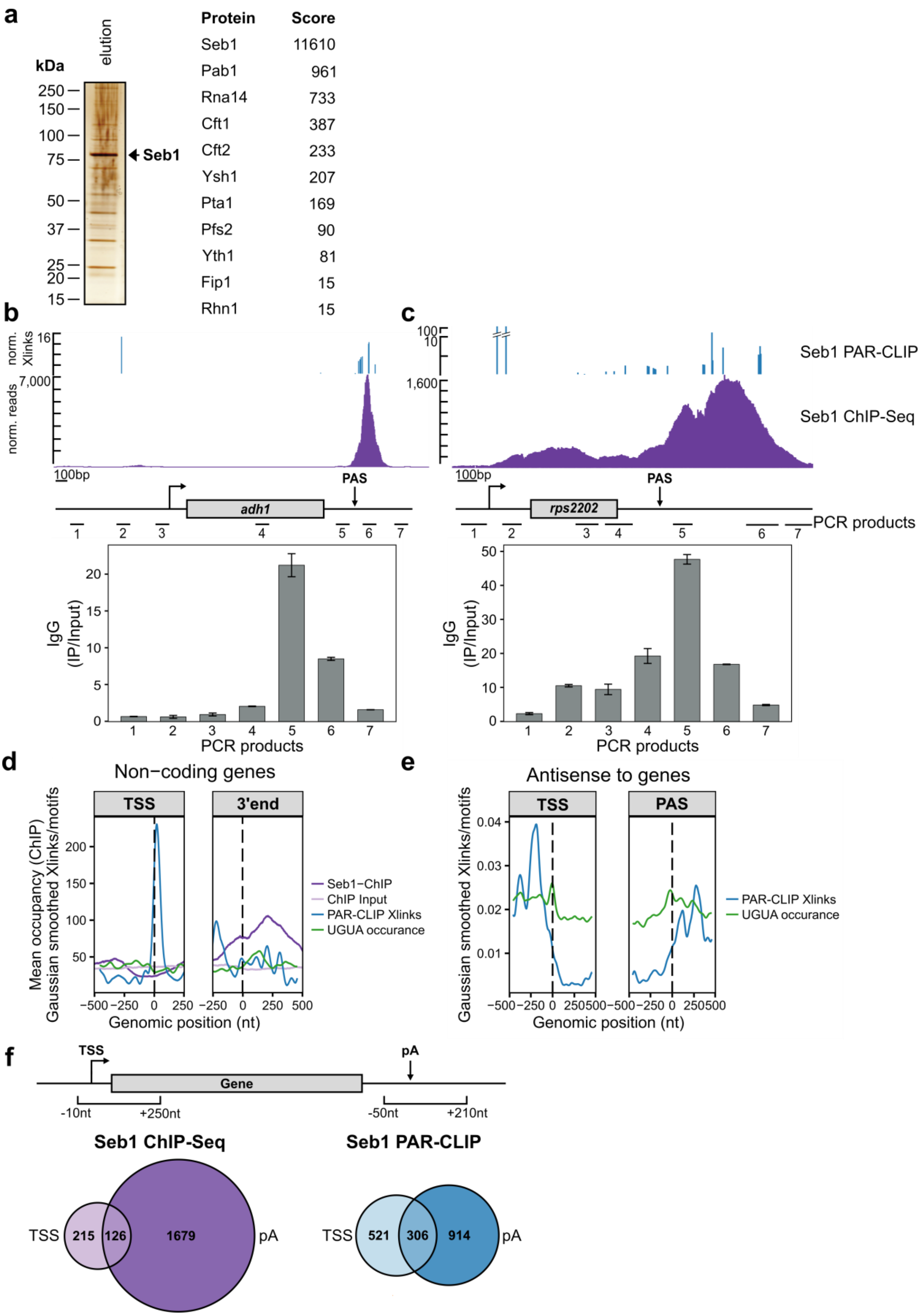
a
elution
kDa
250
150
100
75
50
37
25
20
15
Seb1
Protein Score
Seb1 11610
Pab1 961
Rna14 733
Cft1 387
Cft2 233
Ysh1 207
Pta1 169
Pfs2 90
Yth1 81
Fip1 15
Rhn1 15
b
norm. Xlinks
16
norm. reads
7,000
100bp
PAS
adh1
1 2 3 4 5 6 7
IgG (IP/Input)
PCR products
c
100
10
1,600
Seb1 PAR-CLIP
Seb1 ChIP-Seq
100bp
PAS
rps2202
PCR products
1 2 3 4 5 6 7
IgG (IP/Input)
PCR products
d
Non−coding genes
TSS
3'end
Mean occupancy (ChIP)
Gaussian smoothed Xlinks/motifs
Seb1−ChIP
ChIP Input
PAR-CLIP Xlinks
UGUA occurance
Genomic position (nt)
e
Antisense to genes
TSS
PAS
Gaussian smoothed Xlinks/motifs
PAR-CLIP Xlinks
UGUA occurance
Genomic position (nt)
f
TSS
pA
Gene
-10nt
+250nt
-50nt
+210nt
Seb1 ChIP-Seq
TSS 215 126 1679 pA
Seb1 PAR-CLIP
TSS 521 306 914 pA

Supplementary Figure 1. Seb1 interacts with the CF-CPF complex and is recruited to the 3'end of genes

(a) Silver-stained SDS-PAGE analysis of TEV elution of Seb1-HA-TAP purification. CPF components and termination factors that co-purified with Seb1 as identified by mass spectrometry are listed.

(b) Binding of Seb1 to the *adh1* gene as crosslinks normalized to transcript abundance determined by PAR-CLIP (blue) and as recruitment determined by ChIP-Seq (purple) were visualised using the integrated genome browser (IGB). The schematics below indicates the position relative to the gene. Recruitment to *adh1* was verified using ChIP-qPCR and is shown as a bar plot. The generated PCR products are indicated in the schematics. Error bars indicate standard error of biological duplicates.

(c) Recruitment of Seb1 to the *rps2202* gene as in (b).

(d) Averaged occupancy profiles of Seb1 and input from ChIP, PAR-CLIP crosslinks and occurrence of the Seb1 binding motif UGUA, normalized to transcript levels are shown on non-coding genes. The profiles are aligned to the TSS and PAS as indicated. Genes with a distance less than 250 nt to their neighbouring gene were excluded (n=874). The PAR-CLIP and UGUA motif profiles were smoothed using a Gaussian smoothing function and adjusted to bring to scale with the ChIP-seq profile.

(e) Averaged occupancy profiles of Seb1 from PAR-CLIP crosslinks and occurrence of the UGUA binding motif are shown in antisense direction to annotated genes (n=4,228). The profiles are aligned to the TSS and PAS as indicated. Genes with a distance less than 250 nt to their neighbouring gene were excluded. The ChIP-Seq profiles were not included because of the non-strand-specific nature of the experiment.

(f) Overlap between Seb1 binding to the region 10 nt before to 250 nt after the TSS, and 50 nt before to 210 nt after the PAS as determined by ChIP-Seq (purple) and PAR-CLIP (blue) are shown as Venn diagrams. The same subset of genes was used as in (e).

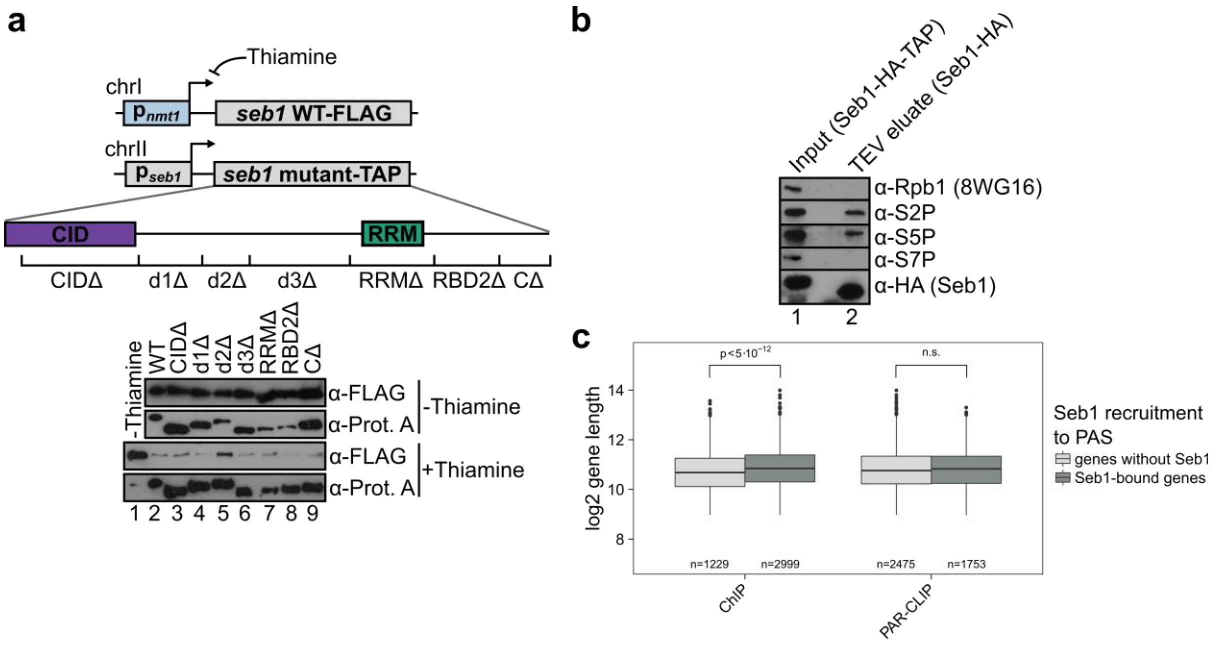

Supplementary Figure 2. Seb1 specifically binds to S2P-Pol II *in vivo*

(a) Western blot showing the expression of Seb1 domain deletion mutants in media containing or lacking thiamine (+ and – thiamine, respectively). The strains used carry two copies of Seb1, a thiamine-repressible WT copy and a mutated version under control of the endogenous promoter. A strain containing two WT copies under control of the different promoters was included as a control. The position of the deletions is indicated in the schematics above the spot test, approximately to scale.

(b) Binding of Seb1-HA-TAP to Pol II in *S. pombe* was determined by immunoprecipitation and TEV elution of Seb1, followed by Western blotting. Differently phosphorylated forms of co-immunoprecipitated Pol II were detected using phospho-specific antibodies as indicated. An antibody that binds to Pol II independently of the phosphorylation state was included as a control (8WG16). Note the change in mobility of Seb1 after TEV cleavage (α-HA).

(c) All genes were split into two different groups: genes that are bound by Seb1 at the PAS ± 250 nt (dark grey) and genes that do not recruit Seb1 (light grey) as determined by either ChIP-Seq (left) or PAR-CLIP (right). The log2 fold gene lengths of these different groups are shown as box plots. The significance of the difference between the groups was calculated using the Wilcoxon-Mann-Whitney test and is indicated above the boxes. The number of genes included in each group is indicated below the boxes. The same subset of genes was used as in Supplementary Fig. 1e.

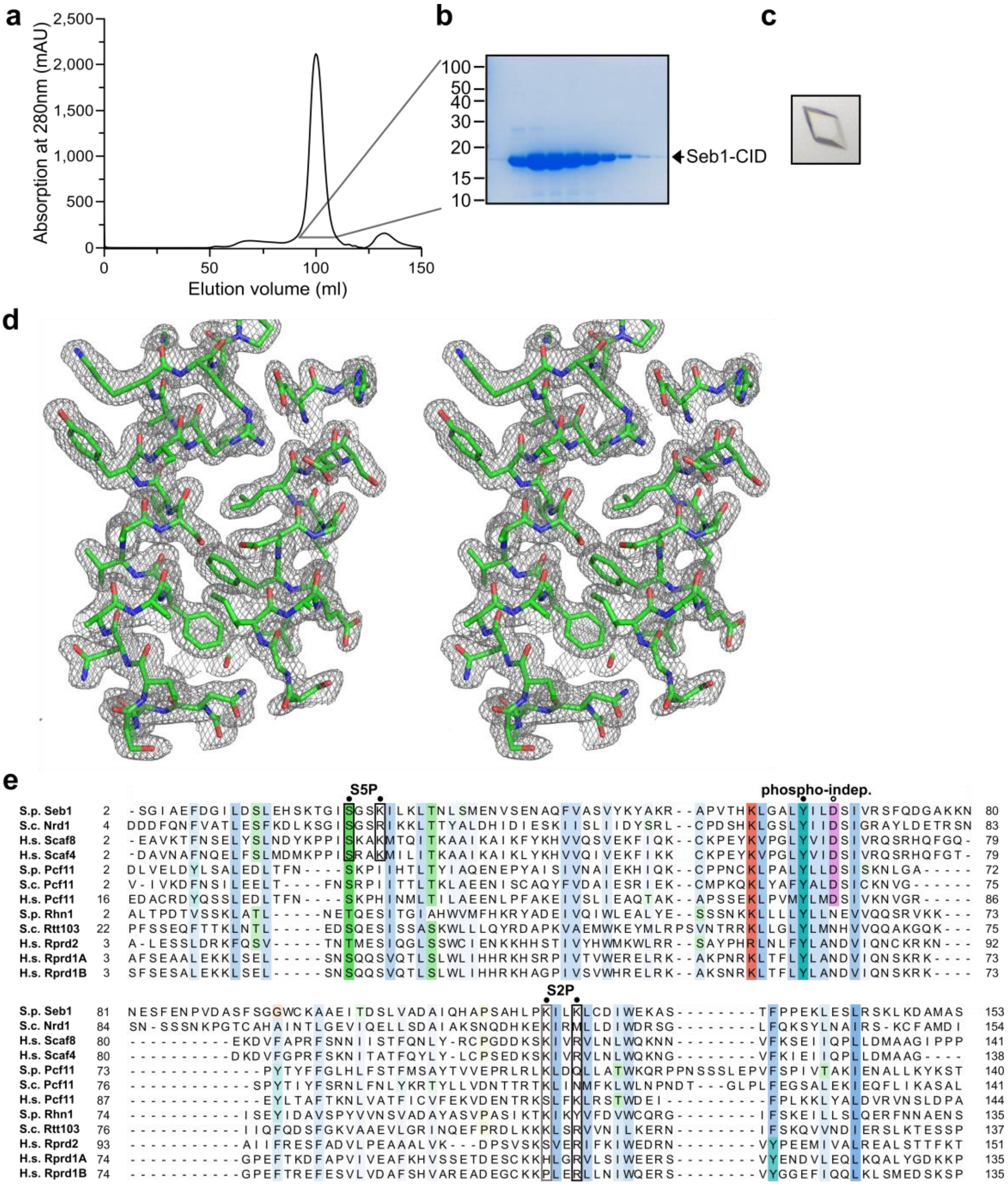

Supplementary Figure 3. Specific amino acids in CIDs recognize phosphorylated CTD peptides

(a) The Seb1-CID$_{1\text{-}152}$-His$_8$ was recombinantly expressed in *E. coli* and subsequently purified using Ni-NTA beads. After elution from the beads by increasing concentrations of imidazole, the protein solution was subject to size exclusion chromatography using a HiLoad Superdex S200 16/60 PrepGrade column (GE Healthcare). The elution of the protein was measured by absorption at 280 nm and is plotted against the elution volume.

(b) The elution fractions from (a) were analysed for the presence of Seb1-CID$_{1\text{-}152}$ by SDS-PAGE as shown.

(c) The protein from (b) was concentrated and used for crystallisation trials. A representative crystal of the CID_{1-152} is shown.

(d) Stereo image of a representative portion of the electron density map of the Seb1-CID_{1-152} crystal. The model is shown as green sticks. The 2Fo-Fc map is shown as a grey mesh and is contoured at 1.0 σ.

(e) Multiple sequence alignment of the CID domains of all proteins shown in Fig. 1a. The conservation of individual amino acids is indicated by colour. Amino acids there were previously shown to be important for S5P or S2P recognition, as well as those which are not expected to bind the CTD in a phospho-specific manner, are indicated by circles, and their conservation in comparison to Seb1 is shown using boxes. Closed circles indicate that the amino acid was mutated in Seb1 *in vitro* and *in vivo*, while open circles indicate analysis by *in vitro* experiments only. The alignment was done using Promals3D and included information about the 3D structures of all CIDs, if known.

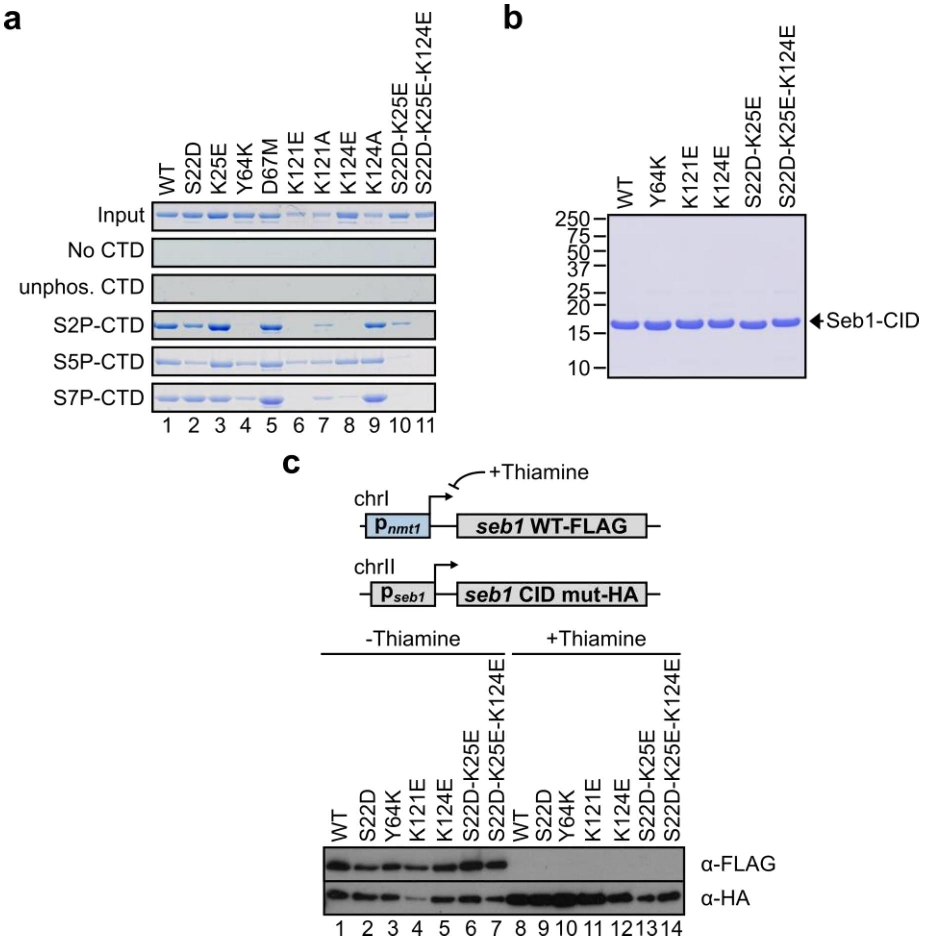

Supplementary Figure 4. Seb1-CID point mutations reduce binding to CTD peptides *in vitro* and *in vivo*

(a) Binding of WT and mutated recombinant full-length Seb1 to CTD peptides was performed as in Fig. 2c and Seb1 binding was analysed by Coomassie stained SDS-PAGE.

(b) SDS-PAGE showing the preparations for the WT Seb-CID$_{1\text{-}152}$ as well as the indicated mutants that were utilised for fluorescence anisotropy (Fig. 2g-i).

(c) Western blot showing expression levels of the indicated Seb1-CID point mutations in cells grown in media containing or lacking thiamine (+ and – thiamine, respectively). The same thiamine-repressible system as in Supplementary Fig. 2a was used and is depicted schematically above the Western blot.

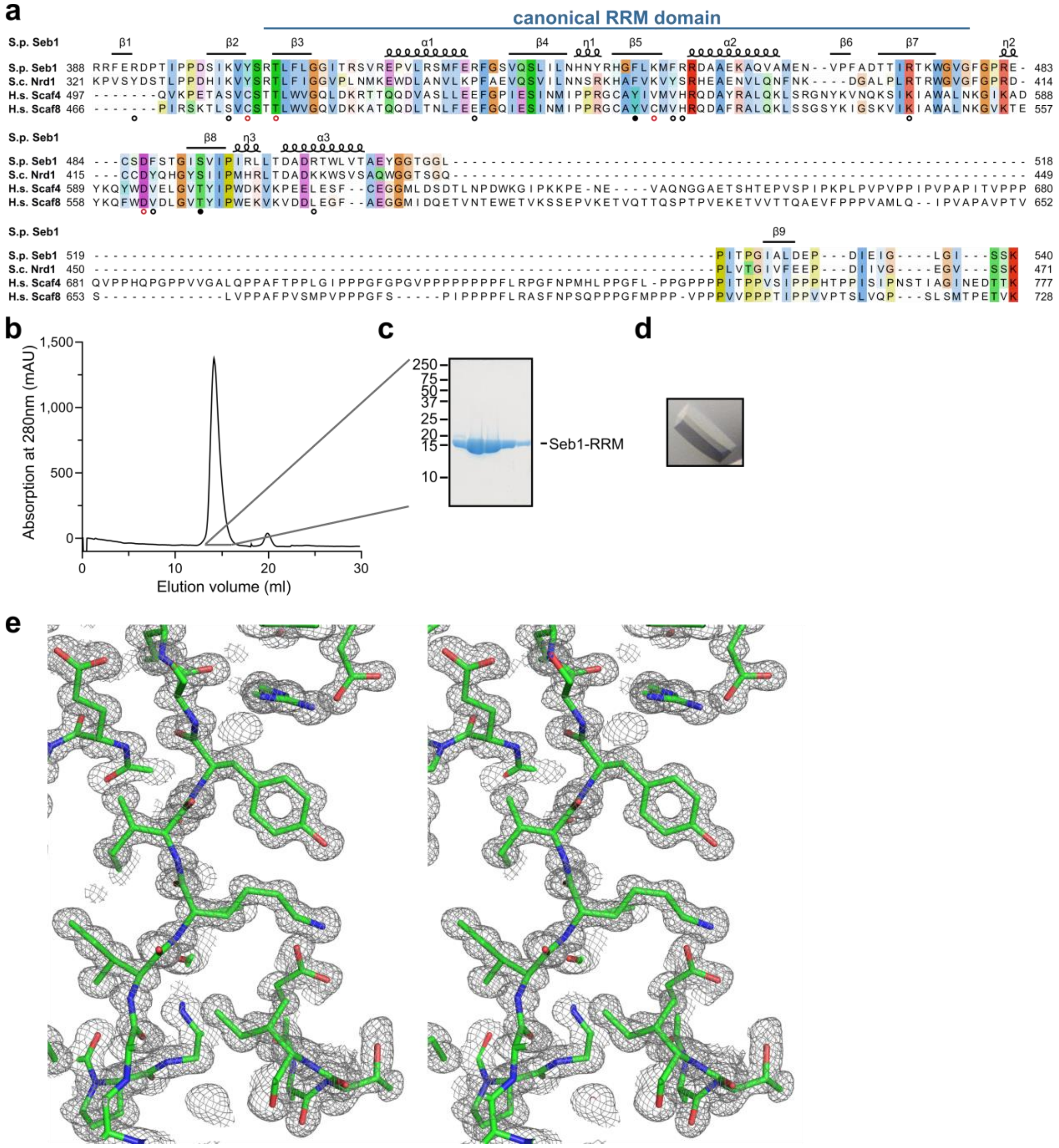

Supplementary Figure 5. The crystal structure of the Seb1-RRM domain shows an unusual conformation

(a) Multiple sequence alignment of RRM domains from indicated CID-proteins. Amino acid conservation is indicated by colour and presence of secondary elements (β sheets or α and η helices) in the Seb1-RRM$_{388\text{-}540}$ structure as well as the canonical RRM region are indicated above the alignment. Amino acids there were mutated are marked by circles. Closed circles mark mutations that were tested *in vitro* and *in vivo*, open circles signify analysis by *in vitro* experiments only, and red circles show amino acid mutations that resulted in insoluble protein.

(b) Seb1-His$_6$-SUMO-RRM$_{388-540}$ was recombinantly expressed in *E. coli* and purified using NiNTA beads. After elution using imidazole, the SUMO tag was cleaved by S3 protease and His$_6$-SUMO and uncleaved protein were removed using NiNTA beads. The flow-through was subjected to size exclusion chromatography using a Superdex 75 10/100 GL column (GE Healthcare). The protein was followed by absorption at 280 nm which is plotted against the elution volume.

(c) Elution fractions from (b) were analysed for the presence of Seb1-RRM$_{388-540}$ by SDS-PAGE.

(d) Protein from (c) was concentrated and used in crystallisation trials. A typical crystal is shown.

(e) Stereo image of a representative portion of the electron density map of the Seb1-RRM$_{388-540}$ crystal. The model is shown as green sticks. The 2Fo-Fc map is shown as a grey mesh and is contoured at 1.0 σ.

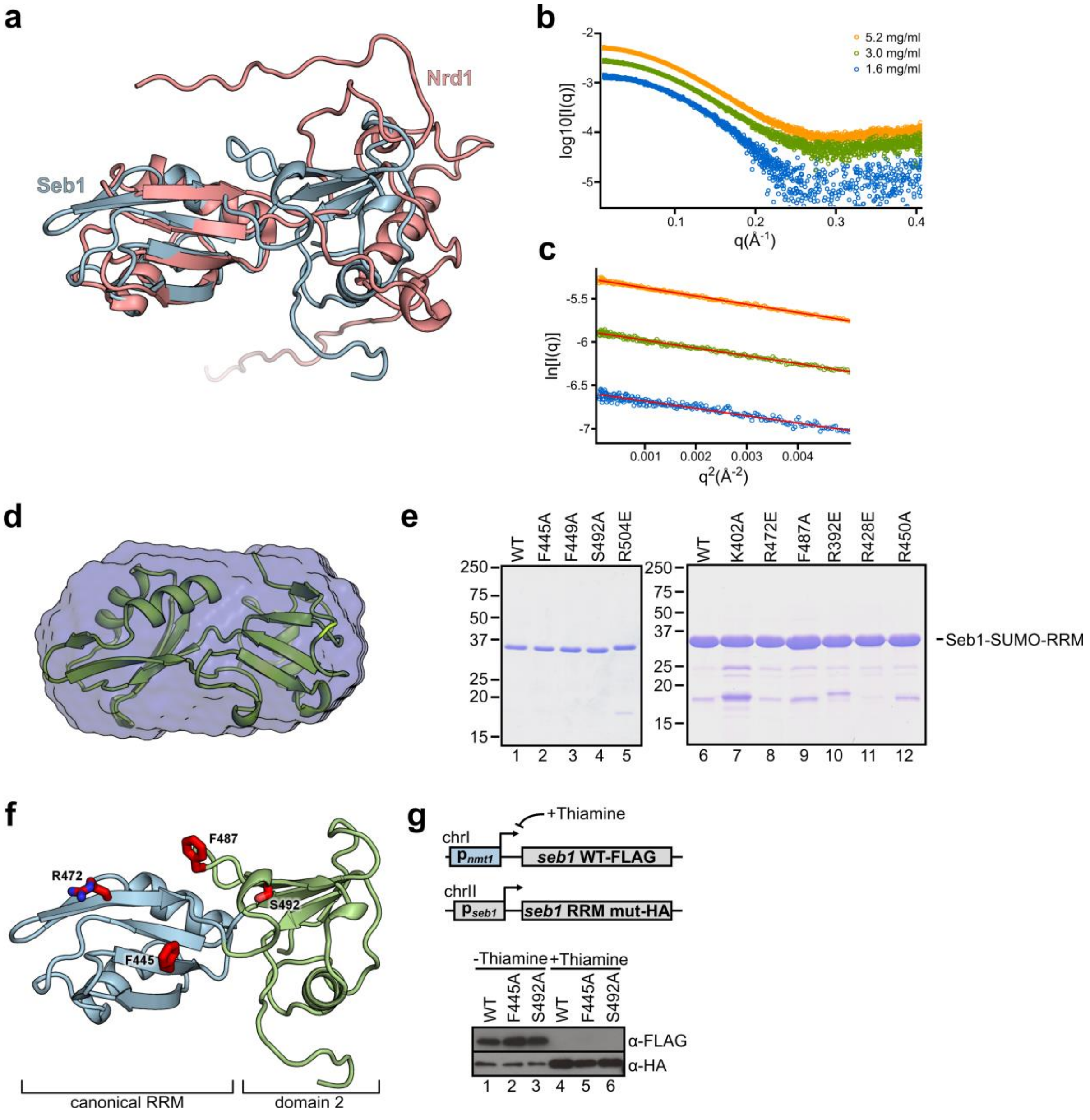
a
Nrd1
Seb1
b
5.2 mg/ml
3.0 mg/ml
1.6 mg/ml
log10[I(q)]
q(Å-1)
c
ln[I(q)]
q2(Å-2)
d
e
WT
F445A
F449A
S492A
R504E
K402A
R472E
F487A
R392E
R428E
R450A
Seb1-SUMO-RRM
f
F487
R472
S492
F445
canonical RRM
domain 2
g
+Thiamine
chrI
seb1 WT-FLAG
chrII
seb1 RRM mut-HA
-Thiamine
α-FLAG
α-HA

Supplementary Figure 6. The conformation of the Seb1-RRM$_{388\text{-}540}$ are identical in solution and in the crystal

(a) The Seb1-RRM$_{388\text{-}540}$ crystal structure (blue) was aligned to the Nrd1-RRM NMR structure (PDBID 2M88, red) using the region of the canonical RRM in both cases.

(b) Plot showing the SAXS curves of the Seb1-RRM$_{388\text{-}540}$ at the three indicated concentrations.

(c) Guinier plots and corresponding fitting (red lines) showing linear behaviour of the SAXS data in the low q region for the three measured concentrations. Sample colouring is as in (e).

(d) Crystal structure of Seb1-RRM$_{388\text{-}540}$ fitted into an *ab initio* bead model calculated from the SAXS data.

(e) SDS-PAGE showing the preparations for the WT Seb-RRM$_{388\text{-}540}$ as well as the different mutants that were utilised for fluorescence anisotropy assays (Fig. 3e).

(f) Structure of the Seb1-RRM$_{388\text{-}540}$ with amino acids highlighted in red that, when mutated, reduce the affinity to RNA as determined in Fig. 3e.

(g) Western blot showing expression of the indicated Seb1-RRM point mutations in *S. pombe* grown in media containing or lacking thiamine (+ and – thiamine, respectively). The same thiamine-repressible system was used as in Fig. 2a and is depicted above the blot.

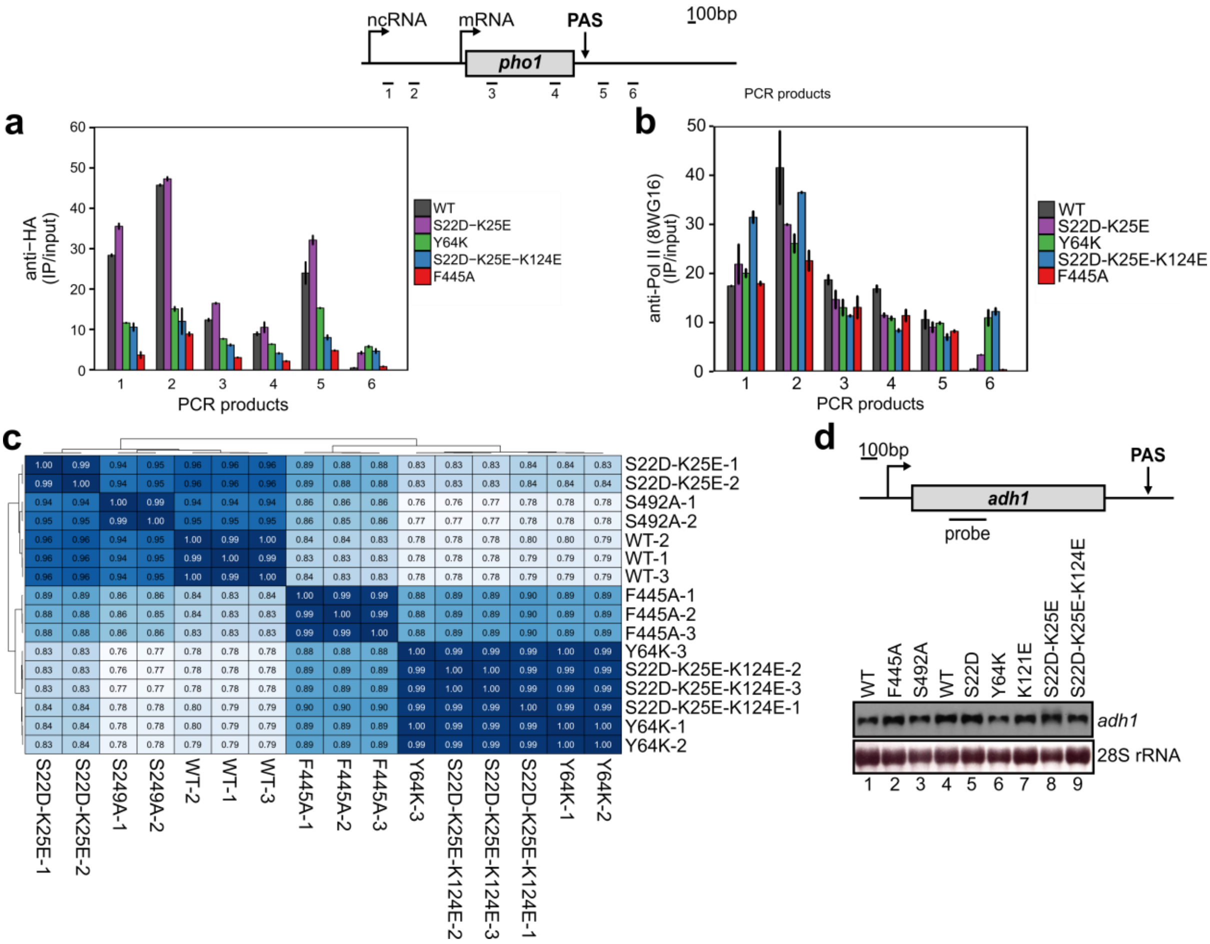

Supplementary Figure 7. Seb1 point mutations cause decreased Seb1 recruitment to *pho1* and no change in *adh1* mRNA abundance

(a) ChIP-qPCR using Seb1-HA with the indicated point mutations was performed to detect recruitment to the *pho1* gene using qPCR primers at positions shown in the schematics above. The same strains as in Figures 2j and 3f were used after depletion of the WT in thiamine-containing medium for 24 hr. Error bars indicate the standard error of biological duplicates.

(b) Same as (a) but antibodies against Pol II (detecting the CTD with the phosphorylation-independent antibody 8WG16) were used.

(c) Spearman correlation matrix between all biological replicates of all strains that were used for RNA-Seq experiments. The bam alignment files were used to calculate the Spearman correlation coefficient after dividing the genome in 100 nt bins, using deepTools version 2.2.2[1].

(d) Strand-specific Northern blot showing expression levels of the *adh1* gene in the indicated mutants after depletion of the WT for 24 hr in thiamine-containing medium (using the same strains as in Fig. 2j and 3f). The position of the probe used relative to the gene is indicated in the schematics above.

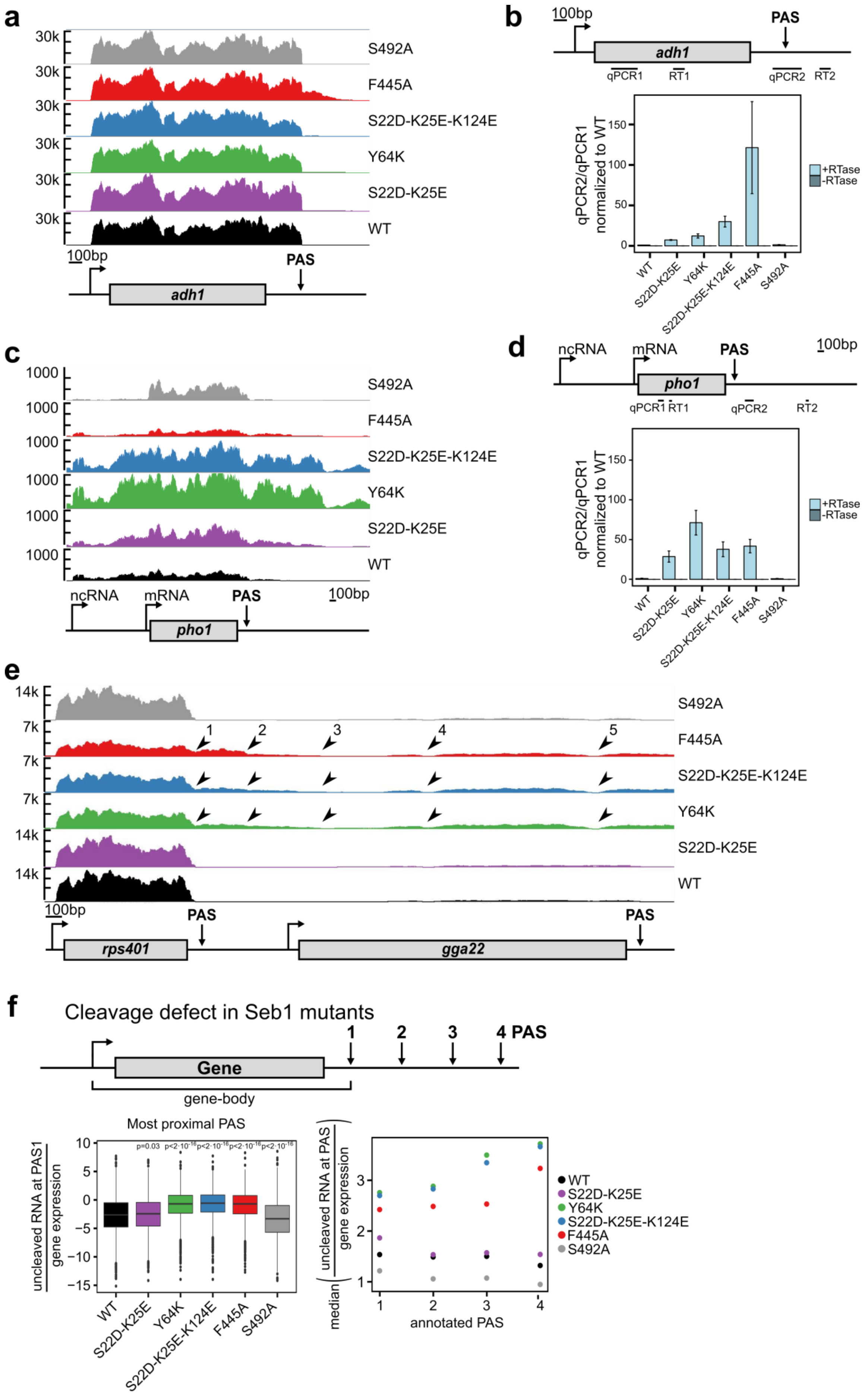
a
S492A
F445A
S22D-K25E-K124E
Y64K
S22D-K25E
WT
30k
100bp
PAS
adh1
b
100bp
PAS
adh1
qPCR1
RT1
qPCR2
RT2
qPCR2/qPCR1 normalized to WT
+RTase
-RTase
c
1000
ncRNA
mRNA
PAS
pho1
d
e
14k
7k
rps401
gga22
f
Cleavage defect in Seb1 mutants
Gene
gene-body
1 2 3 4 PAS
Most proximal PAS
uncleaved RNA at PAS1 / gene expression
median (uncleaved RNA at PAS / gene expression)
annotated PAS
p=0.03
WT
S22D-K25E
Y64K
S22D-K25E-K124E
F445A
S492A

Supplementary Figure 8. Seb1 point mutations show genome-wide read-through *in vivo*

(a) Reads determined by RNA-Seq aligning to the *adh1* gene in the indicated mutants visualised using IGB.

(b) RT-pPCR was used to confirm the read-through visible by RNA-Seq in (a). The position of primers used for RT and regions amplified by qPCR are indicated in the schematics. The read-through signal (qPCR2) was normalized to expression levels (qPCR1) and plotted relative to WT. A control not containing reverse transcriptase (- RTase) was included. Error bars indicate the standard deviation of 3 biological replicates.

(c) Reads aligning to the *pho1* locus as determined by RNA-Seq in the indicated mutants visualised using IGB are shown.

(d) RT-pPCR was used to confirm read-through visible by RNA-Seq in (c). The position of primers used for RT and regions amplified by qPCR are indicated in the schematics above. Data analysis and controls were performed as in (b).

(e) Reads aligning to the *rps401* gene as determined by RNA-Seq in the indicated mutants visualised using IGB are shown. Arrows mark the different PASs observed by Northern blot (Fig. 4d).

(f) The amount of uncleaved RNA in the different point mutants was determined by counting RNA-Seq reads covering the complete length of annotated PAS clusters[2] and normalizing them to gene expression levels (gene-body counts, n=3,914). The boxplot considers only the most proximal PAS clusters if more than one is annotated. The significance of the overall difference between WT and each mutant was determined by the Wilcoxon-Mann-Whitney test and is indicated above each box. For the scatter plot, genes with at least 4 annotated PAS clusters (n=84) were used and the amount of uncleaved reads covering each cluster was averaged after normalization, respectively.

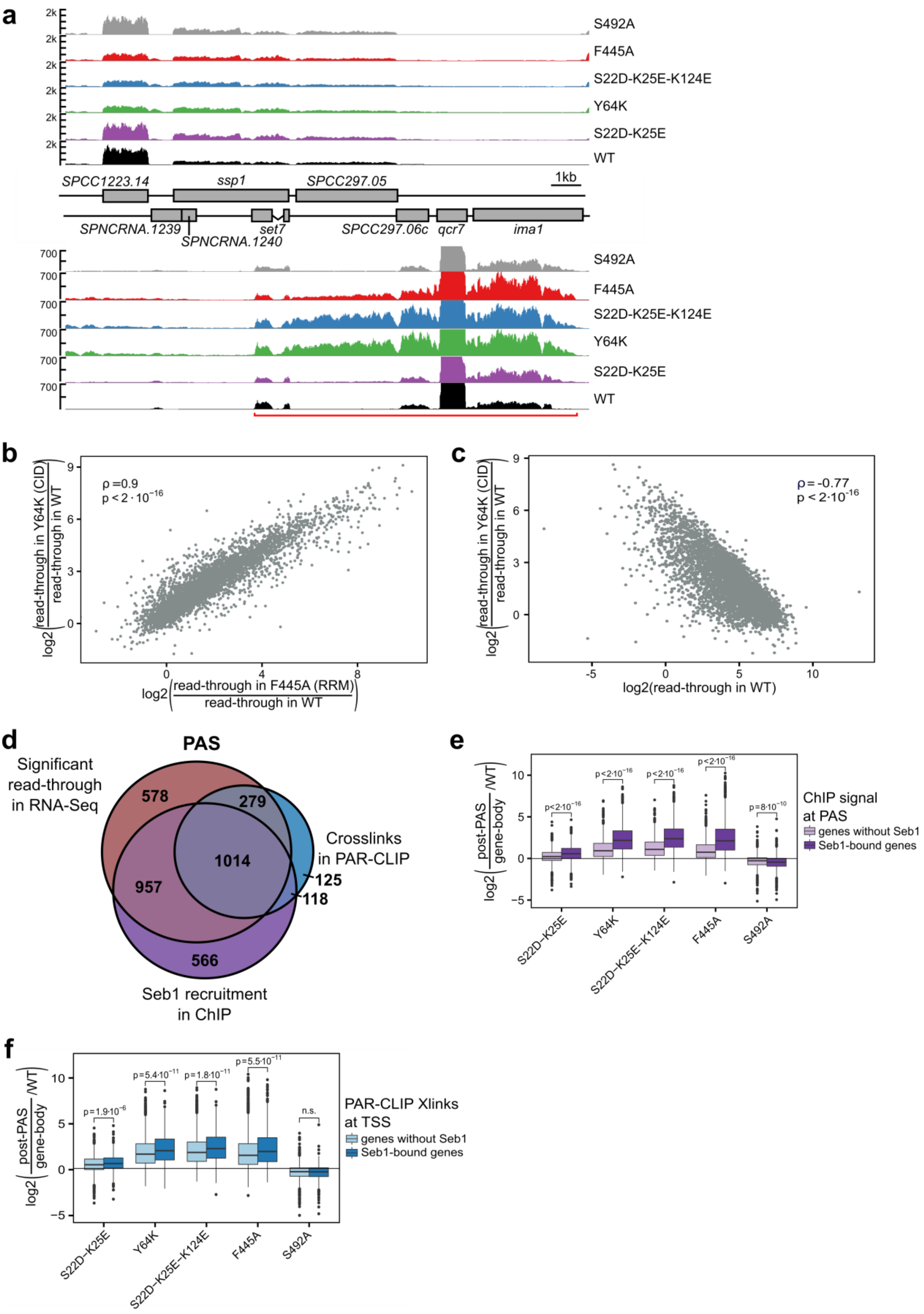
a
S492A
F445A
S22D-K25E-K124E
Y64K
S22D-K25E
WT
SPCC1223.14
ssp1
SPCC297.05
1kb
SPNCRNA.1239
SPNCRNA.1240
set7
SPCC297.06c
qcr7
ima1
b
ρ =0.9
p < 2 · 10^-16
log2(read-through in Y64K (CID) / read-through in WT)
log2(read-through in F445A (RRM) / read-through in WT)
c
ρ = -0.77
p < 2·10^-16
log2(read-through in WT)
d
PAS
Significant read-through in RNA-Seq
578
279
1014
957
125
118
566
Crosslinks in PAR-CLIP
Seb1 recruitment in ChIP
e
log2(post-PAS / gene-body /WT)
ChIP signal at PAS
genes without Seb1
Seb1-bound genes
p=8·10^-10
S22D-K25E
Y64K
S22D-K25E-K124E
F445A
S492A
f
p=1.9·10^-6
p=5.4·10^-11
p=1.8·10^-11
p=5.5·10^-11
n.s.
PAR-CLIP Xlinks at TSS
genes without Seb1
Seb1-bound genes

Supplementary Figure 9. Seb1 CID and RRM work together to ensure correct transcription termination

(a) Reads determined by RNA-Seq aligning to a region on chromosome III in the indicated mutants visualised using IGB.

(b) Scatter plot showing the log2-fold change in read-through levels calculated as in Fig. 4c of the CID mutant Y64K and the RRM mutant F445A. The Spearman correlation coefficient ρ and significance p are shown in the plot.

(c) Scatter plot showing the relation between the log2 fold change in read-through levels in the CID mutant Y64K calculated as in Fig. 4c and log2 basal read-through levels in the WT. The Spearman correlation coefficient ρ and significance p are shown in the plot.

(d) Venn diagram depicting the overlap between genes showing significant read-through in the region 250 nt ± PAS in Seb1 mutants (red) and binding of Seb1 as determined by PAR-CLIP (blue) or ChIP-Seq (purple). The genes defined as having significant read-through in RNA-Seq correspond to the overlap of the three circles in Fig. 4g and therefore show significantly ($p < 0.05$) more read-through than WT in all three mutants, Y64K, S22D-K25E-K124E and F445A. The same subset of genes was used as in Supplementary Fig. 1e.

(e) The log2 fold change in read-through was calculated as in Fig. 4c but in contrast to before, genes were split into two groups, those containing peaks detectable by ChIP-Seq at 250 nt ± PAS (n=2,655) and those that do not (n=1,573). The significance of the overall difference between the two groups of genes was calculated for each mutant by the Wilcoxon-Mann-Whitney test and is indicated above the boxes.

(f) As (e) genes were split according to crosslinks detectable by PAR-CLIP between 10 nt before to 250 nt after the TSS (genes with Xlinks: 827; without Xlinks: 3,401). The significance of the overall difference between the two groups of genes was calculated as in (e) and are indicated above the boxes.

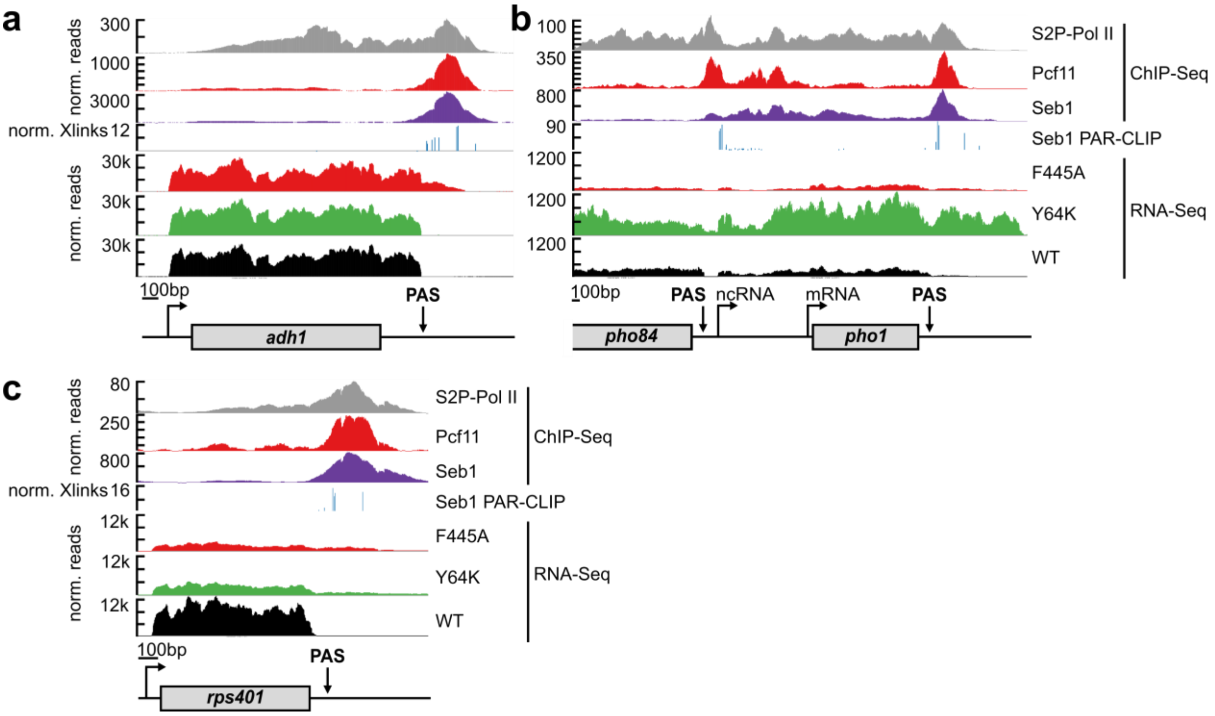

Supplementary Figure 10. Seb1 and Pcf11 binding correlates well with S2P-Pol II levels

(a) Profiles of mapped reads normalized to *adh1* as determined by RNA-Seq of WT, Y64K and F445A after 24 hr in thiamine-containing media, crosslinking sites normalized to transcript abundance from Seb1 PAR-CLIP, and mapped reads normalized to a background control from ChIP-Seq of Seb1-TAP, Pcf11-TAP and S2P-Pol II are shown for the *adh1* gene as indicated.

(b) as (a) but showing the *pho1*, and part of the *pho84,* locus.

(c) as (a) but showing the *rps401* locus.

Figure 2c

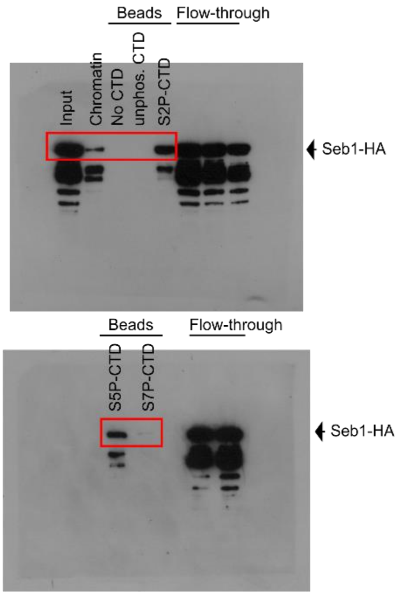

Figure 4d

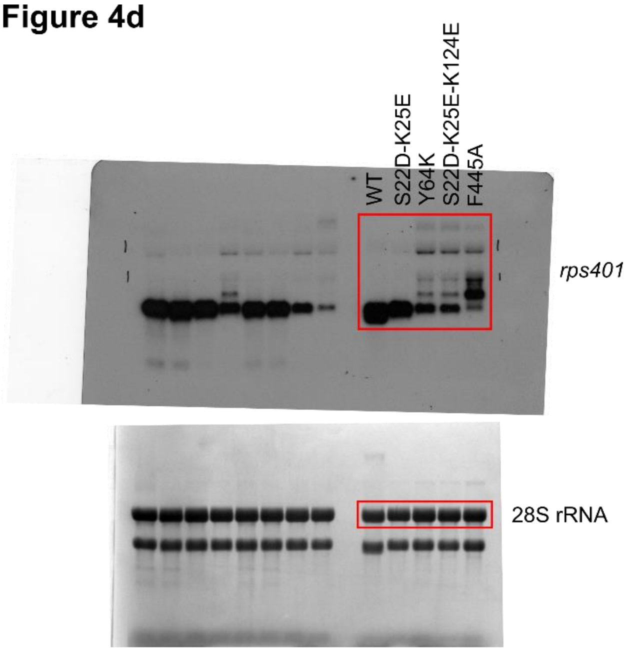

Figure 5e

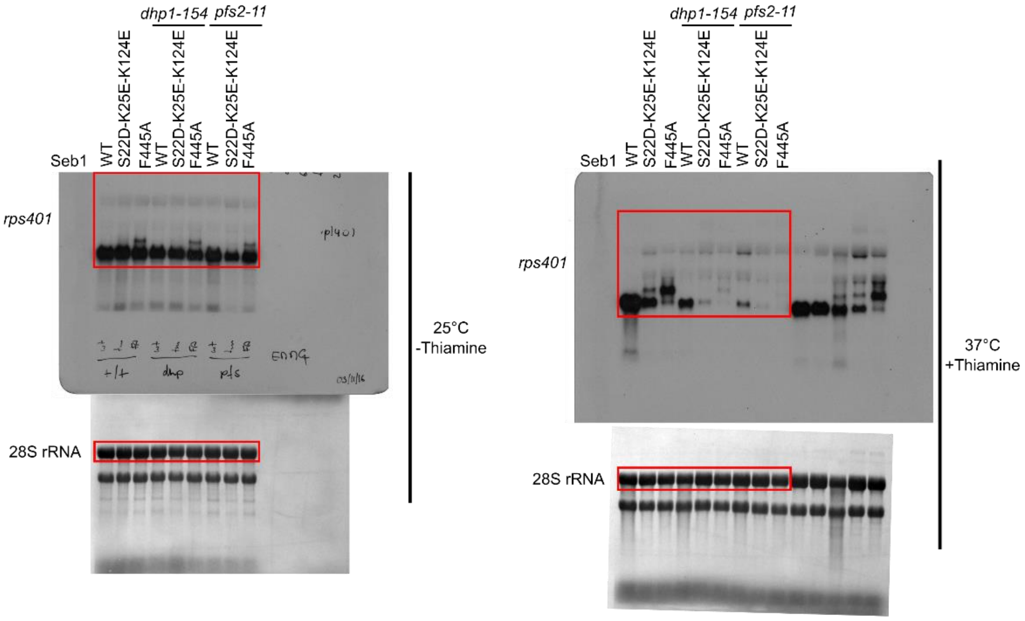

Supplementary Figure 11. Uncropped images of blots shown in the main figures with red boxes indicating cropped regions.

Supplementary Figure 2a

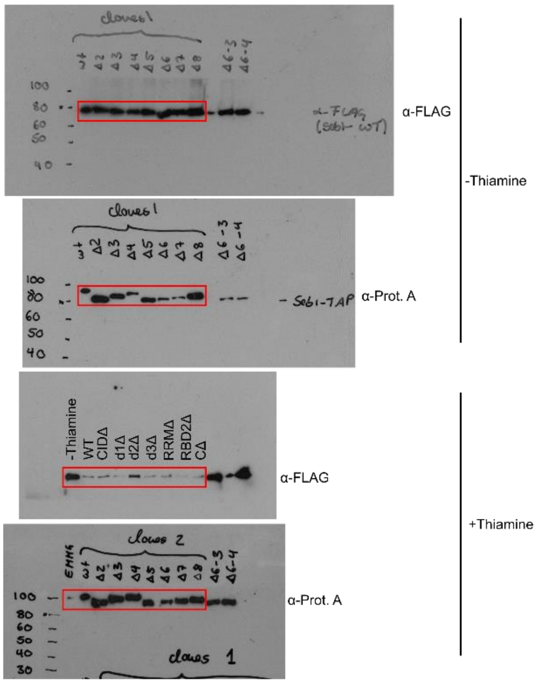

Supplementary Figure 2b

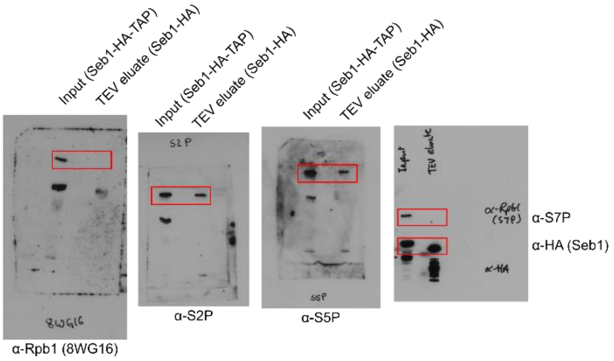

Supplementary Figure 3e

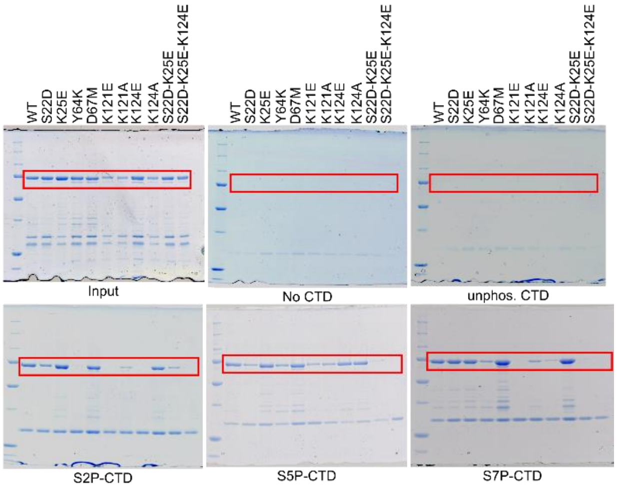

Supplementary Figure 3g

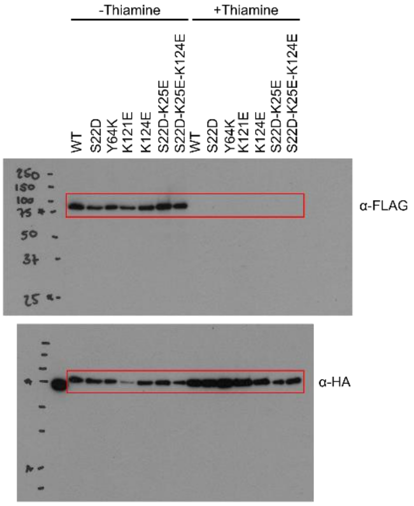

Supplementary Figure 4k

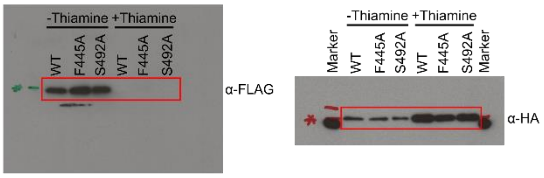

Supplementary Figure 5d

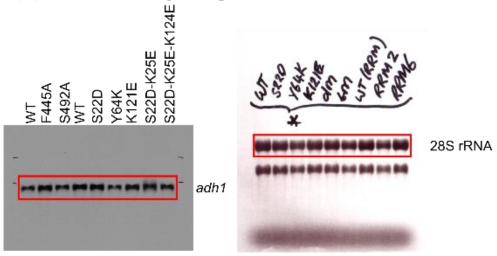

Supplementary Figure 12. Uncropped images of blots and gels shown in supplementary figures with red boxes indicating cropped regions.

Supplementary Table 1. List of *S. pombe* strains

Name	Genotype	Reference
PAR-CLIP	h-, seb1::seb1-TAP::KanMX	generous gift from Damien Hermand
YP21	h+, ura4-294, leu1-32, ade6-M210	Bioneer
YP51	h+, ura4-Δ18, leu1-32, ade6-M216, imr1R(NcoI)::ura4+	[3]
YP144	h+, ura4-Δ18, leu1-32, ade6-M216, his3-Δ1	[4]
YP293	h+, ura4-Δ18, leu1-32, ade6-M210, his3-Δ1	[4]
YP294	h+, ura4-Δ18, leu1-32, ade6-M210, his3-Δ1, pcf11::pcf11-TAP::KanMX	[5]
YP475	h+, ura4-294, leu1-32, ade6-M210, seb1::p(nmt1)-seb1-FLAG::NatMX, leu1::p(seb1)-seb1(F445A)-HA	this study
YP476	h+, ura4-294, leu1-32, ade6-M210, seb1::p(nmt1)-seb1-FLAG::NatMX, leu1::p(seb1)-seb1(S492A)-HA	this study
YP291	h+, ura4-Δ18, leu1-32, ade6-M216, imr1R(NcoI)::ura4+, seb1::seb1-HA-TAP::KanMX	this study
YP510	h+, ura4-294, leu1-32, ade6-M210, seb1::p(nmt1)-seb1-FLAG::NatMX	this study
YP512	h+, ura4-294, leu1-32, ade6-M210, seb1::p(nmt1)-seb1-FLAG::NatMX, leu1::p(seb1)-seb1-TAP	this study
YP531	h+, ura4-294, leu1-32, ade6-M210, seb1::p(nmt1)-seb1-FLAG::NatMX, leu1::p(seb1)-seb1(Δ2-152)-TAP	this study
YP532	h+, ura4-294, leu1-32, ade6-M210, seb1::p(nmt1)-seb1-FLAG::NatMX, leu1::p(seb1)-seb1(Δ153-223)-TAP	this study
YP533	h+, ura4-294, leu1-32, ade6-M210, seb1::p(nmt1)-seb1-FLAG::NatMX, leu1::p(seb1)-seb1(Δ224-278)-TAP	this study
YP534	h+, ura4-294, leu1-32, ade6-M210, seb1::p(nmt1)-seb1-FLAG::NatMX, leu1::p(seb1)-seb1(Δ279-391)-TAP	this study
YP535	h+, ura4-294, leu1-32, ade6-M210, seb1::p(nmt1)-seb1-FLAG::NatMX, leu1::p(seb1)-seb1(Δ392-488)-TAP	this study
YP536	h+, ura4-294, leu1-32, ade6-M210, seb1::p(nmt1)-seb1-FLAG::NatMX, leu1::p(seb1)-seb1(Δ489-562)-TAP	this study
YP537	h+, ura4-294, leu1-32, ade6-M210, seb1::p(nmt1)-seb1-FLAG::NatMX, leu1::p(seb1)-seb1(Δ563-620)-TAP	this study
YP539	h+, ura4-294, leu1-32, ade6-M210, seb1::seb1-FLAG::NatMX	this study
YP587	h+, ura4-294, leu1-32, ade6-M210, seb1::p(nmt1)-seb1-FLAG::NatMX, leu1::p(seb1)-seb1-HA	this study
YP588	h+, ura4-294, leu1-32, ade6-M210, seb1::p(nmt1)-seb1-FLAG::NatMX, leu1::p(seb1)-seb1(S22D)-HA	this study
YP589	h+, ura4-294, leu1-32, ade6-M210, seb1::p(nmt1)-seb1-FLAG::NatMX, leu1::p(seb1)-seb1(Y64K)-HA	this study
YP590	h+, ura4-294, leu1-32, ade6-M210, seb1::p(nmt1)-seb1-FLAG::NatMX, leu1::p(seb1)-seb1(K121E)-HA	this study
YP591	h+, ura4-294, leu1-32, ade6-M210, seb1::p(nmt1)-seb1-FLAG::NatMX, leu1::p(seb1)-seb1(S22D-K25E)-HA	this study

YP592	h+, ura4-294, leu1-32, ade6-M210, seb1::p(nmt1)-seb1-FLAG::NatMX, leu1::p(seb1)-seb1(S22D-K25E-K124E)-HA	this study
YP804	h unknown, ura4 unknown; leu1 unknown; ade6 unknown; ura4::p(nmt1)-seb1-FLAG::NatMX; leu1::seb1-S22D-K25E-K124E-HA, dhp1-154	this study, based on [6]
YP811	h unknown, ura4 unknown; leu1 unknown; ade6 unknown; ura4::p(nmt1)-seb1-FLAG::NatMX; leu1::seb1-(WT)-HA, dhp1-154	this study, based on [6]
YP812	h unknown, ura4 unknown; leu1 unknown; ade6 unknown; ura4::p(nmt1)-seb1-FLAG::NatMX; leu1::seb1-S22D-K25E-K124E-HA, pfs2-11	this study, based on [7]
YP819	h unknown, ura4 unknown; leu1 unknown; ade6 unknown; ura4::p(nmt1)-seb1-FLAG::NatMX; leu1::seb1-F445A-HA, dhp1-154	this study, based on [7]
YP820	h unknown, ura4 unknown; leu1 unknown; ade6 unknown; ura4::p(nmt1)-seb1-FLAG::NatMX; leu1::seb1-(WT)-HA, pfs2-11	this study, based on [7]
YP821	h unknown, ura4 unknown; leu1 unknown; ade6 unknown; ura4::p(nmt1)-seb1-FLAG::NatMX; leu1::seb1-F445A-HA, pfs2-11	this study, based on [7]

Supplementary Table 2. List of oligonucleotides

Number	Name	Sequence	Purpose
2469	adh1-1 fwd	CGGAAGCTGGTGAGAAGAAC	qPCR
2470	adh1-1 rev	CGTTGGAATGCGGAGTAGAG	qPCR
2471	adh1-2 fwd	CAACCTCCCATTTCCTCCTT	qPCR
2472	adh1-2 rev	GTGGACACATTTCGGGAATC	qPCR
2473	adh1-3 fwd	TCTCTCGCTTTCCTCATTCG	qPCR
2474	adh1-3 rev	GCCAACTGCTTGTCAGGAAT	qPCR
2475	adh1-4 fwd	GGTCCCGAGAACGTCAAGT	qPCR, Northern probe
2476	adh1-4 rev	ACTTGACACCAACACGGTCA	qPCR, Northern probe
2478	adh1 gene body	AATGGCAACAACACGCATAG	RT
2536	pho1 gene body	CAAACATACCATATCCATACCAAAGAG	RT
2547	pho1-5 fwd	AGCAGAGGATAGTTTATGTAGGAGATAATG	qPCR
2548	pho1-5 rev	TTTATATGGTGAGAGTATTGTCAAAGAAAC	qPCR
2857	pho1-6 rev	AAACTAAGTCTTGACAACTATAACGAAACC	qPCR
2857	pho1 read-through	AAACTAAGTCTTGACAACTATAACGAAACC	RT
3048	pho1-1 fwd	ACAATTATATCTTGGTCTGGGGAAC	qPCR

3049	pho1-1 rev	ATCATTAAATTGTGAATATCGCAAG AC	qPCR
3050	pho1-2 fwd	ATGTTTGAGATTTACGGGAAGTG	qPCR
3051	pho1-2 rev	TTTGTCCTAATTTTCCAAACAGC	qPCR
3052	pho1-3 fwd	TTTGTACCAACTTGGACTCCTG	qPCR
3053	pho1-3 rev	GCGTCCCATGTCAAATAACTC	qPCR
3054	pho1-4 fwd	CTTCGCCTTTACTCATGATGC	qPCR
3055	pho1-4 rev	TTGGTAGGAAGTAGGCAATGG	qPCR
3089	adh1-5 fwd	GTACGACGATCCCTAATCCAAC	qPCR
3090	adh1-5 rev	ACGCAAATCTTGAAAAAGATCC	qPCR
3129	pho1-6 fwd	AAAATTCTATGTTTCTATACATGCCT CTG	qPCR
3422	Seb1 L3	AAGATTTTGCTATGCGTCGT	cloning
3423	Seb1 L4	TAATTAACCCGGGGATCCGTCGACC TTGGGGTTGCCAAGGAGGTT	cloning
3424	Seb1 L5	AAACGAGCTCGAATTCATCGATGAT ATGTGTTAAAA	cloning
3425	Seb1 L6	TCGCGATTTGATCTTTTTG	cloning
3620	adh1-6 fwd	CGAAAACGAAGCGCTTTACTC	qPCR
3621	adh1-6 rev	TCACTTTGCCATTCATCTGTCT	qPCR
3622	adh1-7 fwd	ATGCAACGTTGTGCAGTGAT	qPCR
3623	adh1-7 rev	CAGTCCATTTGTGCGTACGT	qPCR
3778	rps2202-1 fwd	CCGTATATGCCCTTCAGGTT	qPCR
3828	Seb1-NdeI-fwd	TAATTACATAATGTCGGGAATCGCT GAATTC	cloning
3829	Seb1-NotI-rev	TTATATGCGGCCGCTTGGGGTTGCC AAG	cloning
3831	Seb1 rev	TTAATTGGGGTTGCCAAGGAG	cloning
3878	K25E fwd	GGATCAGAAATTTTGAAATTGACTA ACTTGTCG	site-directed mutagenesis
3879	K25E rev	AATTTCAAAATTTCTGATCCCGAGA TTCCTG	site-directed mutagenesis
3881	K22D fwd dmut	CAAGACAGGAATCGACGGATCAGA AATTTT	site-directed mutagenesis
3882	K22D rev	TGATCCGTCGATTCCTGTCTTGGAAT GTT	site-directed mutagenesis
3883	Y64K fwd	GGAGCTTTGAAATTCTAGACTCAAT CGTTCGTAG	site-directed mutagenesis
3884	Y64K rev	GTCTAGAATTTTCAAAGCTCCTAATT TATGGGTGAC	site-directed mutagenesis
3885	D66M fwd	GTATATTCTAATGTCAATCGTTCGTA GCTTTCAGG	site-directed mutagenesis
3886	D66M rev	CGATTGACATTAGAATATACAAAGC TCCTAATTTATG	site-directed mutagenesis
3887	K124E fwd	GCCTAAAATATTAGAACTTTGTGAT ATTTGGGAGAAG	site-directed mutagenesis
3888	K124E rev	CAAATATCACAAAGTTCTAATATTT TAGGCAAATGAGC	site-directed mutagenesis
3895	rps2202-1 rev	TGTATCTACAGGAGCAGTCACA	qPCR

3898	rps2202-3 fwd	CTGAACGGCCGTATCAACAA	qPCR
3899	rps2202-3 rev	ACAATCACACCAACTTGACGA	qPCR
3900	rps2202-4 fwd	TGTCCCATAATGAGGCTCGT	qPCR
3901	rps2202-4 rev	GCCAGCCTTTTCACCGTGA	qPCR
3902	rps2202-5 fwd	ACTTAGTCTCTGGTTTCGAGCA	qPCR
3903	rps2202-5 rev	TCAACGCCTCTCTCACTTCT	qPCR
3932	rps2202-6 fwd	ACTCTGGCACTGTCTGAAGA	qPCR
3933	rps2202-6 rev	TACTCTTCTACGGCGGCATT	qPCR
3934	rps2202-7 fwd	CGCTGATATGACTTGTGTACAGT	qPCR
3935	rps2202-7 rev	ACCGATTCCCATTTTGTGCT	qPCR
4027	rps2202-2 fwd	ACAAGATGTGAGCGGAAGTC	qPCR
4028	rps2202-2 rev	CGAGAAGCGCGTTAGTTTC	qPCR
4088	after CID delete rev 180	ACCAACATAACCACCATTCCC	cloning
4089	after CID delete fwd 278	GGATCGGTCAATGATACCCAGAG	cloning
4090	before RRM delete fwd 392	TGTCTTCATCCCCATGGGACC	cloning
4091	before RRM delete rev 280	CGTGACCCAACCATTCCACC	cloning
4092	RRM delete rev 392	CTCAAATCGGCGAGGAAATCC	cloning
4093	RRM delete short fwd 488	ACAGGAATCAGCGTTATCCCAATC	cloning
4094	RRM delete long fwd 562	TATAGGGGAGGTCCACCCATTC	cloning
4095	C delete rev 563	TGGTTTACGACCCCGGAATCG	cloning
4096	C delete TAP fwd 620	GGTCGACGGATCCCCGG	cloning
4105	K124A fwd	GCCTAAAATATTAGCACTTTGTGAT ATTTGGGAGAAG	site-directed mutagenesis
4106	K124A rev	CAAATATCACAAAGTGCTAATATTT TAGGCAAATGAGC	site-directed mutagenesis
4107	K121A fwd	TGCTCATTTGCCTGCAATATTAAAG CTTTG	site-directed mutagenesis
4108	K121A rev	CTTTAATATTGCAGGCAAATGAGCA GATGG	site-directed mutagenesis
4109	K121E fwd	TGCTCATTTGCCTGAAATATTAAAG CTTTG	site-directed mutagenesis
4110	K121E rev	CTTTAATATTTCAGGCAAATGAGCA GATGG	site-directed mutagenesis
4118	Seb1-AdcI-fwd	ATAAGGCGCGCCGAACCAAATGCAC GAGTA	cloning
4252	Seb1-XhoI-152 rev	TATATACTCGAGTGCCATTGCATCTT TCAGC	cloning
4359	CID delete fwd 154	AGTACGGAACCGGTTAGTGTAGATT C	cloning
4360	after CID delete1 rev 152	TGCCATTGCATCTTTCAGCTTT	cloning

4361	after CID delete1 fwd 224	CCTGCCGTCGCACCATCC	cloning
4362	after CID delete2 rev 223	CTGCGGTGGAGTGCTAACTG	cloning
4363	RRM delete second rev 488	GGAAAAGTCAGAGCACTCTCGAG	cloning
4444	Seb1 L4-FLAG-pFa	CCGGGGATCCGTCGACCCCTACTTG TCATCGTCATCCTTGTAGTCGATGTC ATGATCTTTATAATCACCGTCATGGT CTTTGTAGTCTCCACCCCCGCCTCCC CCTTGGGGTTGCCAAGG	cloning
4524	Seb1 F445A fwd	GACACGGGGCCTTGAAAATGTTTC	site-directed mutagenesis
4525	Seb1 F445A rev	CATTTTCAAGGCCCCGTGTCTGTA	site-directed mutagenesis
4532	Seb1 S492A fwd	ACAGGAATCGCCGTTATCCCAATCC	site-directed mutagenesis
4533	Seb1 S492A rev	GGGATAACGGCGATTCCTGTGG	site-directed mutagenesis
4591	Seb1 KpnI R388 fwd	TAATTAGGTACCCGCCGATTTGAGC GTGAC	cloning
4592	Seb1 HindIII K540 rev	TTATATAAGCTTACTTAGAACTTATT CCTAATCCAATTTC	cloning
4676	ProtA_del_fwd	TGAGGCGCGCCACTTCTAA	cloning
4677	ProtA_del_rev	AGCGTAATCTGGAACGTCATATG	cloning
5074	HA-AscI-rev	TATAGGCGCGCCTCAAGCGTAATCT GGAAC	cloning
5240	adh1 read-through	ACTTTGACGCTATAAGACATGCA	RT
5508	rps401-1F	TGGAAAACTGGTACGTCCAAA	qPCR
5509	rps401-1R	AGAATATCGATGCCGAGTGC	qPCR
5510	rps401-2F	CACCAAAAATGGTTCGAGGT	qPCR
5511	rps401-2R	ATCAAAGGAAGGCACTCACG	qPCR
5512	rps401-3F	CTTTGAACGGACGTGAGGTT	qPCR
5513	rps401-3R	TTCAACGGAGATCACATCCA	qPCR
5514	rps401-4F	GATCAAGGTCAACGACACCA	qPCR
5515	rps401-4R	ACGACCACCGGTAACCATAA	qPCR
5522	rps401-8F	CAACCAAAAAGGCTACGTGAA	qPCR
5523	rps401-8R	GGTAGACGTCCAATTTCGTCA	qPCR
5548	rps401-5bF	TGCTTTGGACCGTGAGTTTG	qPCR
5549	rps401-5bR	GAGCTTGACACCCTTACCCT	qPCR
5550	rps401-6bF	AGATATGTCGCGTGCATAAGT	qPCR
5551	rps401-6bR	GCTAACAAACCTCTCCAACTGG	qPCR
5552	rps401-7bF	ACAAAACAATTTCCTACGAGCG	qPCR
5553	rps401-7bR	ACCTGCCAACAATGTGACTC	qPCR

Supplementary Table 3: List of plasmids used in this study

Number	Description	Purpose
3932	pET41a(+)-Seb1(full-length)-His8	recombinant expression
3934	pET41a(+)-Seb1(S22D)-His8	recombinant expression
3935	pET41a(+)-Seb1(K25E)-His8	recombinant expression
3936	pET41a(+)-Seb1(Y64K)-His_8	recombinant expression
3937	pET41a(+)-Seb1(D67M)-His_8	recombinant expression
3938	pET41a(+)-Seb1(K124E)-His_8	recombinant expression
3939	pET41a(+)-Seb1(S22D-K25E)-His_8	recombinant expression
3940	pET41a(+)-Seb1(S22D-K25E-K124E)-His_8	recombinant expression
3943	pET41a(+)-Seb1(K124A)-His_8	recombinant expression
3944	pET41a(+)-Seb1(K121E)-His_8	recombinant expression
3945	pET41a(+)-Seb1(K121A)-His_8	recombinant expression
3967	pOPINS3C-SUMO-His_6-Seb1(388-540)	recombinant expression
3981	pDUAL-p(seb1)-seb1(Δ2-152)-TAP	expression in *S. pombe*
3982	pDUAL-p(seb1)-seb1(Δ153-223)-TAP	expression in *S. pombe*
3983	pDUAL-p(seb1)-seb1(Δ224-278)-TAP	expression in *S. pombe*
3984	pDUAL-p(seb1)-seb1(Δ279-391)-TAP	expression in *S. pombe*
3985	pDUAL-p(seb1)-seb1(Δ392-488)-TAP	expression in *S. pombe*
3986	pDUAL-p(seb1)-seb1(Δ489-562)-TAP	expression in *S. pombe*
3987	pDUAL-p(seb1)-seb1(Δ563-620)-TAP	expression in *S. pombe*
4028	pDUAL-p(seb1)-seb1-HA	expression in *S. pombe*
4029	pDUAL-p(seb1)-seb1(S22D)-HA	expression in *S. pombe*
4030	pDUAL-p(seb1)-seb1(Y64K)-HA	expression in *S. pombe*
4031	pDUAL-p(seb1)-seb1(K121E)-HA	expression in *S. pombe*
4032	pDUAL-p(seb1)-seb1(S22D-K25E)-HA	expression in *S. pombe*
4033	pDUAL-p(seb1)-seb1(S22D-K25E-K124E)-HA	expression in *S. pombe*
4041	pOPINS3C-SUMO-His_6-Seb1(388-540)-T407A	recombinant expression
4042	pOPINS3C-SUMO-His_6-Seb1(388-540)-F445A	recombinant expression
4043	pOPINS3C-SUMO-His_6-Seb1(388-540)-K447D	recombinant expression
4044	pOPINS3C-SUMO-His_6-Seb1(388-540)-F449A	recombinant expression
4045	pOPINS3C-SUMO-His_6-Seb1(388-540)-D486K	recombinant expression
4046	pOPINS3C-SUMO-His_6-Seb1(388-540)-S492A	recombinant expression
4047	pOPINS3C-SUMO-His_6-Seb1(388-540)-R504E	recombinant expression

4055	pOPINS3C-SUMO-His_6-Seb1(388-540)-K447A	recombinant expression
4056	pOPINS3C-SUMO-His_6-Seb1(388-540)-D486A	recombinant expression
4062	pDUAL-p(seb1)-seb1(F445A)-HA	expression in *S. pombe*
4063	pDUAL-p(seb1)-seb1(S492A)-HA	expression in *S. pombe*
4065	pOPINS3C-SUMO-His_6-Seb1(388-540)-K402A	recombinant expression
4066	pOPINS3C-SUMO-His_6-Seb1(388-540)-Y404A	recombinant expression
4068	pOPINS3C-SUMO-His_6-Seb1(388-540)-R472E	recombinant expression
4071	pOPINS3C-SUMO-His_6-Seb1(388-540)-F479A	recombinant expression
4072	pOPINS3C-SUMO-His_6-Seb1(388-540)-F487A	recombinant expression
4083	pET41a(+)-Seb1(1-152)-His_8	recombinant expression
4096	pDUAL-p(seb1)-seb1(K124E)-HA	expression in *S. pombe*
4104	pET41a(+)-Seb1(1-152)-Y64K-His_8	recombinant expression
4105	pET41a(+)-Seb1(1-152)-K121E-His_8	recombinant expression
4106	pET41a(+)-Seb1(1-152)-K124E-His_8	recombinant expression
4107	pET41a(+)-Seb1(1-152)-S22D-K25E-His_8	recombinant expression
4108	pET41a(+)-Seb1(1-152)-S22D-K25E-K124E-His_8	recombinant expression

Supplementary Methods

Crystallisation and data collection

Crystallisation trials were carried out in a sitting-drop vapour diffusion format in 96-well Greiner plates at 20.5°C using a Cartesian Technologies robotic pipetting system[8]. We obtained diamond-shaped crystals of the Seb1-CID$_{1\text{-}152}$ in a condition containing 100 mM Tris, pH 8.0 and 4 M NaCl after one day. Crystals were cryo-protected with 25% (v/v) glycerol and flash-cooled in liquid nitrogen. Diffraction data were collected at 100 K on the beamline I03 at Diamond Light Source (DLS), Didcot, UK. Data were processed with XIA2[9] in the space group $P3_121$ (Table 2).

Crystallisation screening of Seb1-RRM$_{388\text{-}540}$ was performed as above and initial crystals grew after one day using mother liquor containing 1 M ammonium formate, 100 mM sodium cacodylate, pH 6.5 and 8% (w/v) poly- γ-glutamic acid polymer (PGA-LM, 200-400 kDa low molecular weight polymer). As before, 25% (v/v) glycerol was used for cryo-protection and crystals were flash-cooled in liquid nitrogen. Native diffraction data were collected at 100 K on the beamline I04 at DLS and processed using XIA2 in the space group C121. For sulphur single-wavelength anomalous dispersion (S-SAD) datasets were recorded from 22 RRM$_{388\text{-}540}$ crystals at a wavelength of 1.77 Å using the inverse beam method (5° wedges) on beamline I03 at DLS.

Structure determination and refinement

The structure of Seb1-CID$_{1\text{-}152}$ was phased via molecular replacement with PHASER[10] using the CID domain of Nrd1 as a search model (PDBID: 3CLJ). Model building and refinement were performed iteratively using COOT[11] and AUTOBUSTER[12] with TLS parameters. The quality of the final geometry was assessed with MolProbity[13]. A total of 98.7% of residues were in the Ramachandran favoured region and 1.3% were in the Ramachandran allowed region. A stereo image of a portion of the electron density map is shown in Supplementary Fig. 3d.

For Seb1-RRM$_{388\text{-}540}$ phases were obtained experimentally using S-SAD. Datasets from 16 crystals were merged using XIA2 and sulphur sites were located with HKL2MAP[14]. After iterative main-chain tracing with SHELXE, density modification and phase extension to native resolution (1.0 Å) we generated an initial model with PHENIX autobuild[15]. Manual model building with COOT and refinement with PHENIX refine were carried out iteratively. In the final rounds we performed anisotropic atomic displacement parameter (ADP) refinement.

Secondary structure elements were assigned using PHENIX ksdssp, model geometry was assessed with MolProbity. A total of 97.5% of residues were in the Ramachandran favoured region and 2.5% were in the Ramachandran allowed region. A stereo image of a portion of the electron density map is shown in Supplementary Fig. 5e.

Small angle X-ray scattering

Small angle X-ray scattering (SAXS) data were collected at beamline B21 at DLS at 16°C using a sample to detector distance of 3.9 m and a wavelength of 1 Å. Buffer subtraction, data merging and subsequent analysis were performed using ScÅtter (www.bioisis.net/scatter). Fitting of the crystal structure of Seb1-$RRM_{388-540}$ to the SAXS curve was carried out using the FoXS webserver[16]. The pair distribution function P(r) was determined within ScÅtter and twenty-three independent *ab initio* bead models were calculated using DAMMIF[17], averaged with DAMAVER and finally a refinement run with DAMMIN[18] was performed. The crystal structure was fitted into the resulting *ab initio* model using the program SUPCOMB.

Genome-wide data analysis

For ChIP-Seq, the resulting sequences were trimmed to remove low quality reads (less than Phred score 20) and reads shorter than 20 nt using Trimmomatic (version 0.36)[19]. Reads were subsequently aligned to the *S. pombe* genome (ASM294v2.28) using Bowtie2 version 2.2.6[20]. Peak calling was done with MACS2 2.1.1.20160309[21] and used to define binding in indicated regions. Metagene profiles were generated by calculating the mean of all aligned reads for each base pair in the indicated window using the indicated set of genes with R.

For PAR-CLIP, adapter sequences are first trimmed from the raw sequencing files. The quality filter then discards all reads containing unidentified nucleotides, Phred scores below 30, reads shorter than 15 nt, or reads that are flagged by Illumina's internal chastity filter. Quality-trimmed reads are aligned to the S. pombe genome (ASM294v2.25 from Pombase.org) using the short read aligner Bowtie (version 1.1.1-)[22] with a maximum of one mismatch and taking unique matches only (options: -q -p 16 -S -nohead -v 1 -e 70 -l 28 -y -a -m 1 -best -strata -phred33 -quals). The resulting SAM files were converted into BAM and Pileup format using SAMTools[23].

For RNA-Seq, reads were trimmed by quality (less than Phred score 20) and reads shorter than 20 nt were removed using Trimmomatic (Galaxy Version 0.32.3)[19]. The resulting reads were aligned to the *S. pombe* genome (ASM294v2.28) using TopHat (Galaxy version 0.9)[24]. Data analysis was performed with R using in-house scripts featuring Bioconductor packages[25,26].

Supplementary References

1. Ramirez, F., Dundar, F., Diehl, S., Gruning, B.A. & Manke, T. deepTools: a flexible platform for exploring deep-sequencing data. *Nucleic Acids Res* **42**, W187-91 (2014).
2. Mata, J. Genome-wide mapping of polyadenylation sites in fission yeast reveals widespread alternative polyadenylation. *RNA Biol* **10**, 1407-14 (2013).
3. Buhler, M., Spies, N., Bartel, D.P. & Moazed, D. TRAMP-mediated RNA surveillance prevents spurious entry of RNAs into the Schizosaccharomyces pombe siRNA pathway. *Nat Struct Mol Biol* **15**, 1015-23 (2008).
4. Lemieux, C. et al. A Pre-mRNA degradation pathway that selectively targets intron-containing genes requires the nuclear poly(A)-binding protein. *Molecular cell* **44**, 108-119 (2011).
5. Lemay, J.F. et al. The Nrd1-like protein Seb1 coordinates cotranscriptional 3' end processing and polyadenylation site selection. *Genes Dev* **30**, 1558-72 (2016).
6. A, J.M., Gaudioso-Pedraza, R. & Benitez-Alfonso, Y. Callose deposition and symplastic connectivity are regulated prior to lateral root emergence. *Commun Integr Biol* **6**, e26531 (2013).
7. Wang, S.W., Asakawa, K., Win, T.Z., Toda, T. & Norbury, C.J. Inactivation of the pre-mRNA cleavage and polyadenylation factor Pfs2 in fission yeast causes lethal cell cycle defects. *Mol Cell Biol* **25**, 2288-96 (2005).
8. Walter, T.S. et al. A procedure for setting up high-throughput nanolitre crystallization experiments. Crystallization workflow for initial screening, automated storage, imaging and optimization. *Acta Crystallogr D Biol Crystallogr* **61**, 651-7 (2005).
9. Winter, G., Lobley, C.M. & Prince, S.M. Decision making in xia2. *Acta Crystallogr D Biol Crystallogr* **69**, 1260-73 (2013).
10. McCoy, A.J. et al. Phaser crystallographic software. *J Appl Crystallogr* **40**, 658-674 (2007).
11. Emsley, P. & Cowtan, K. Coot: model-building tools for molecular graphics. *Acta Crystallogr D Biol Crystallogr* **60**, 2126-32 (2004).
12. Smart, O.S. et al. Exploiting structure similarity in refinement: automated NCS and target-structure restraints in BUSTER. *Acta Crystallogr D Biol Crystallogr* **68**, 368-80 (2012).
13. Davis, I.W. et al. MolProbity: all-atom contacts and structure validation for proteins and nucleic acids. *Nucleic Acids Res* **35**, W375-83 (2007).
14. Pape, T. & Schneider, T.R. HKL2MAP: a graphical user interface for macromolecular phasing with SHELX programs. *Journal of Applied Crystallography* **37**, 843-844 (2004).
15. Adams, P.D. et al. The Phenix software for automated determination of macromolecular structures. *Methods* **55**, 94-106 (2011).
16. Schneidman-Duhovny, D., Hammel, M., Tainer, J.A. & Sali, A. FoXS, FoXSDock and MultiFoXS: Single-state and multi-state structural modeling of proteins and their complexes based on SAXS profiles. *Nucleic Acids Res* (2016).
17. Van Le, B. et al. Structural and functional characterization of soluble endoglin receptor. *Biochem Biophys Res Commun* **383**, 386-91 (2009).
18. Svergun, D.I. Restoring low resolution structure of biological macromolecules from solution scattering using simulated annealing. *Biophys J* **76**, 2879-86 (1999).
19. Bolger, A.M., Lohse, M. & Usadel, B. Trimmomatic: a flexible trimmer for Illumina sequence data. *Bioinformatics* **30**, 2114-20 (2014).
20. Langmead, B. & Salzberg, S.L. Fast gapped-read alignment with Bowtie 2. *Nat Methods* **9**, 357-9 (2012).

21. Zhang, Y. et al. Model-based analysis of ChIP-Seq (MACS). *Genome Biol* **9**, R137 (2008).
22. Langmead, B., Trapnell, C., Pop, M. & Salzberg, S.L. Ultrafast and memory-efficient alignment of short DNA sequences to the human genome. *Genome Biol* **10**, R25 (2009).
23. Li, H. et al. The Sequence Alignment/Map format and SAMtools. *Bioinformatics* **25**, 2078-9 (2009).
24. Kim, D. et al. TopHat2: accurate alignment of transcriptomes in the presence of insertions, deletions and gene fusions. *Genome Biol* **14**, R36 (2013).
25. Huber, W. et al. Orchestrating high-throughput genomic analysis with Bioconductor. *Nat Methods* **12**, 115-21 (2015).
26. Gentleman, R.C. et al. Bioconductor: open software development for computational biology and bioinformatics. *Genome Biol* **5**, R80 (2004).
